# Provision of Therapeutic Hypothermia in Neonatal Transport: A Longitudinal Study and Review of Literature

**DOI:** 10.7759/cureus.270

**Published:** 2015-05-26

**Authors:** Alok Sharma

**Affiliations:** 1 Neonatal Medicine and Surgery, University Hospital Southampton

**Keywords:** therapeutic hypothermia, cooling, hypothermia

## Abstract

Background: Worldwide, a significant proportion of infants needing therapeutic hypothermia for hypoxia-ischaemia are transported to a higher-level facility for neonatal intensive care. They pose technical challenges to transport teams in cooling them. Concerns exist about the efficacy of passive cooling in neonatal transport to achieve a neurotherapeutic temprature. Servo-controlled cooling in the standard of care on the neonatal unit. The key question is whether the same standard of care in the neonatal unit can be safely used for therapeutic hypothermia during transport of neonates with suspected hypoxia-ischaemia.

Methods: A prospective cross-sectional survey of United Kingdom (UK) neonatal transport services (n=21) was performed annually from 2011-2014 with a 100% response. The survey ascertained information about service provision and the method of cooling used during transport.

Results: In 2011, all UK neonatal transport services provided therapeutic hypothermia during transport. Servo-control cooling machines were used by only 6 of the 21 teams (30%) while passive cooling was used by 15 of the 21 (70%) teams. In 2012 9 of the 21 teams (43%) were using servo-control. By 2014 the number of teams using servo-control cooling had more than doubled to 15 of the 21 (62%) services. Teams have done this through modification of transport trolleys and dedicated ambulances.

Conclusion: Servo-controlled cooling in neonatal transport is becoming more common in the UK. The question remains whether it should be endorsed as a standard of care. Some teams continue to passively cool neonates with hypoxia-ischaemia during transport. This article reviews the drivers, current evidence, safety and processes involved in provision of therapeutic hypothermia during neonatal transport to enable teams to decide what would be the right option for them.

## Introduction

Events in the perinatal period severe enough to cause neonatal hypoxic-ischemic encephalopathy (HIE) occur in 3 per 1000 births in the UK [1]. Therapeutic hypothermia is neuroprotective and should be implemented as soon as possible after birth [2-3].

The British Association of Perinatal Medicine recommends the transfer of such neonates to centres with experience of providing therapeutic hypothermia. Transport teams should be capable of doing so round the clock [4].

A significant proportion of infants benefiting from therapeutic hypothermia is born outside centres equipped to provide it. In a study conducted by Kendall, et al., 39 neonates were referred to the London neonatal transport service from 18 hospitals for transfer to one of eight specialist cooling centres over a nine-month period [5]. In East of England between October 2009 and October 2010, 68% of neonates transported were outborn [6]. Based on various studies, we estimate the proportion of encephalopathic infants requiring transfer for cooling varies from 50% to 73% [5-8].

The transfer process involves different cooling practices, including passive cooling, servo-controlled cooling, and use of adjuncts. Adjuncts used in passive cooling include gel packs, ice, and fans [5-10].

The efficacy of passive, active, and servo-controlled cooling in neonatal transport have been evaluated in a number of studies [5-14]. The Whole-Body Hypothermia for the Treatment of Perinatal Asphyxial Encephalopathy (TOBY) group has endorsed passive cooling during neonatal transport [15]. There are concerns about whether passive cooling is effective and that it may cause overcooling [6-8]. On the other hand, active cooling with gel packs or ice can cause significant overcooling as well as injury to the skin and fat necrosis [5-7, 10-11]. There are currently no evidence-based guidelines for the provision of therapeutic hypothermia in neonatal transport. The TOBY group has helped standardise the delivery of passive cooling in neonatal transport [15]. Servo-controlled active cooling has evolved over the years, albeit without standardisation of the equipment or the process. This article looks at the evolution in the provision of cooling during neonatal transport over a four-year period. It helps to provide neonatal transport teams with evidence-based information with regards to how they might want to provide therapeutic hypothermia in their respective regions worldwide. 

## Materials and methods

This study was a cross-sectional evaluation of the protocols of all neonatal transport services (n=21) in the United Kingdom; it was conducted in November 2011. Transport services were identified by the Neonatal Transport Interest Group service-leads database. Each transport service was sent a paper survey to be completed by a consultant or nurse. There was 100% response. The collected information included the cooling method used during transport, the guidelines followed, the mode of temperature monitoring, and whether the temperatures during transfer were audited. Where the use of servo-controlled cooling during transport was reported, the method used to secure the machine was obtained. A telephone call was placed to each service, and additional information was collected regarding the use of a 24-hour service and the presence/absence of a dedicated ambulance. Longitudinal follow-up was performed through telephone survey for all transport services in December 2012, 2013, and 2014, as a significant proportion of services were moving from passive cooling to servo-controlled cooling in neonatal transport. The factors involved in the decision making were evaluated.

No funding was needed. An ethical review was not sought, and no incentives were offered for completion of the survey.

## Results

Responses were received from all the neonatal transport services across the UK. All transport services offered facilities to transport neonates requiring therapeutic hypothermia during the study period. At the time of the initial survey in 2011, 18 of the 21 (85%) neonatal transport services were operating a 24-hour service for the transport of such neonates (Table 1).

**Table 1:** Service Provision for Therapeutic Hypothermia by Neonatal Transport Teams United Kingdom (2011-2014)

Year	2011	2012	2013	2014
Passive cooling	15/21	12/21	8/21	6/21
Servo-control	6/21	9/21	13/21	15/21
Servo-control with dedicated ambulance	5/21	8/21	12/21	14/21
24/7 service	18/21	18/21	19/21	19/21
Audit	21/21	21/21	21/21	21/21
Rectal temp	21/21	21/21	21/21	21/21
Outreach education	17/21	19/21	21/21	21/21

**Figure 1:**
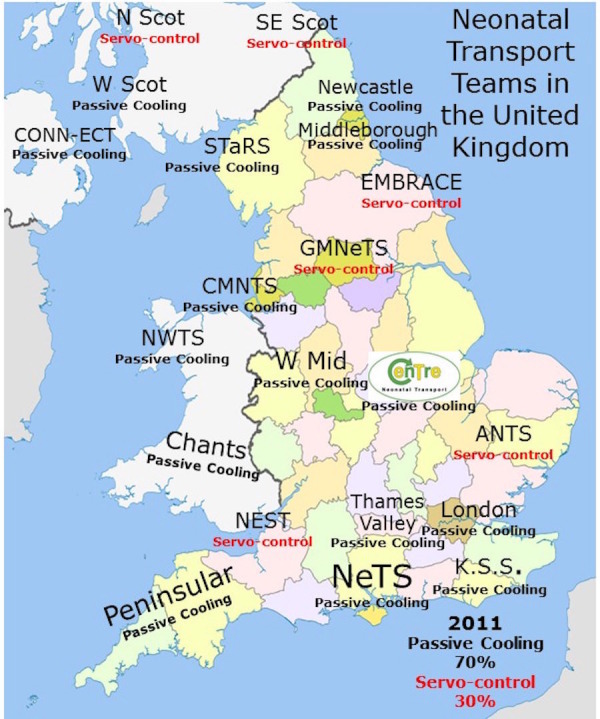
Transport Teams United Kingdom 2011 Cooling methodology used by each transport team in the UK

**Figure 2:**
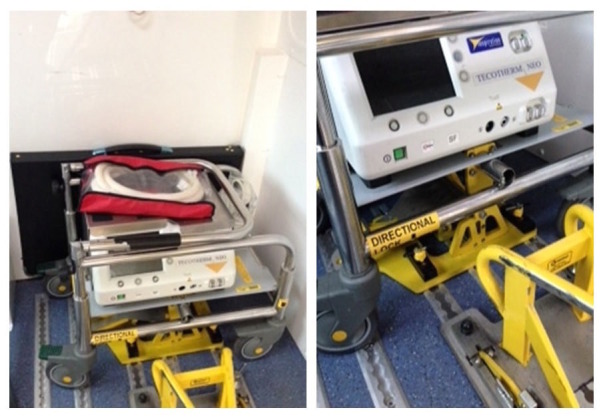
Tecotherm Machine in Dedicated Ambulance Tecotherm machine secured separate from transport trolley (Photographs courtesy West Midlands Neonatal Transport Team Birmingham United Kingdom)

**Figure 3:**
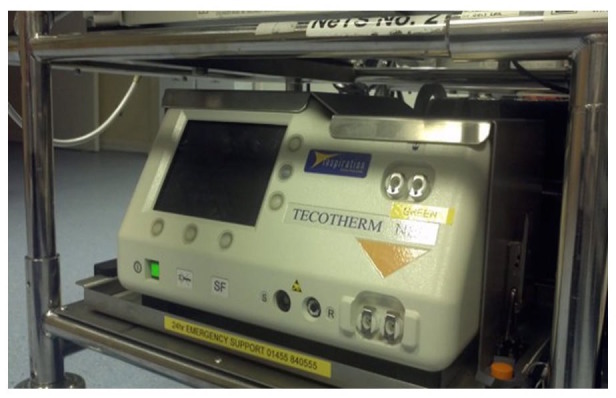
Tecotherm Machine in Transport Trolley Tecotherm servo-control cooling machine in secure box on transport trolley

**Figure 4:**
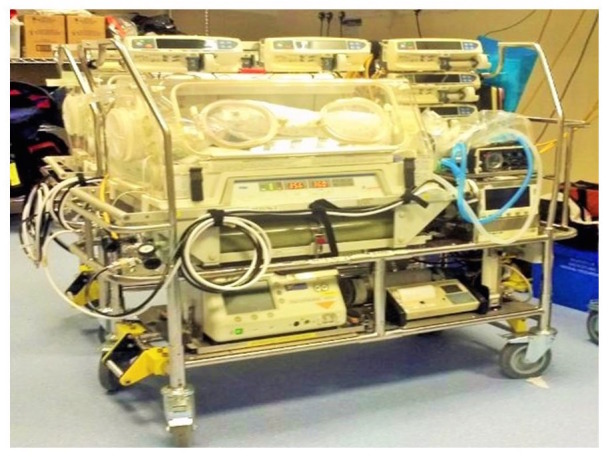
Transport Trolley with Secure Cooling Machine This trolley can be secured in any front-line ambulance with a Falcon 6 base

**Figure 5:**
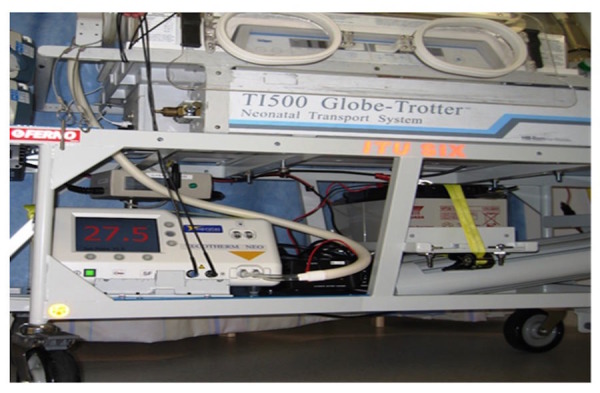
Power Source Battery source for the cooling machine secured on the trolley (Photograph courtesy Greater Manchester Neonatal Transport Team Manchester United Kingdom)

Passive cooling was used by 15 of the 21 (70%) neonatal transport teams. These teams used a passive cooling transport guideline produced by the TOBY group [13]. Servo-controlled equipment for cooling was used by six out of the 21 (30%) neonatal transport services (Figure 1). Teams using servo-controlled cooling did so either through the modification of a dedicated ambulance to secure the cooling machine to a ramp in the ambulance (Figure 2) or a modification of their existing transport trolley to secure the cooling machine in a specialised area on it (Figures 3-5). All but one of the six (83%) transport services providing cooling with a servo-controlled machine used dedicated ambulances in 2011. All used rectal probes to monitor temperature during cooling. Nineteen of the 21 (94%) neonatal transport teams completed audits of temperature control for this group of patients. Fourteen of the 21 (66%) neonatal transport services had a dedicated ambulance, yet only five of 14 (35%) used servo-control cooling during the actual transport process. Approximately half of all services providing passive cooling were considering the use of servo-controlled machines for the purpose of providing cooling during transport at the time of the initial survey in December 2011. On asking them why they had not, the lack of a dedicated ambulance was thought to be a major factor.

Outreach education or other educational events on hypothermia were organised by 17 of the 21 (81%) neonatal transport services within their networks. This assisted in increasing awareness of the need for the early recognition of neonates who met the cooling criteria to enable the prompt transfer of such babies.

In the repeat survey performed in December 2012, a further three neonatal transport services were using servo-controlled therapeutic hypothermia, bringing the total to nine out of 21 (43%). All of these services had dedicated ambulances. Outreach education was carried out by 19 of the 21 transport services (90.5%).

**Figure 6:**
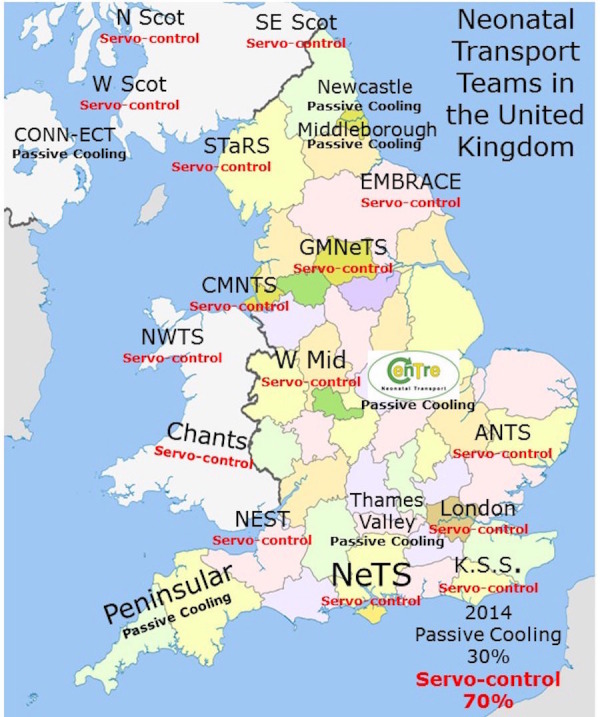
Transport Teams United Kingdom 2014 Cooling methodology used by each transport team in the UK

When the survey was repeated in December 2013 and 2014 (Table 1), the number of transport services delivering servo-control during neonatal transport had continued to increase. All of the services contemplating moving to servo-control had completed the move. In 2014, 14 out of 21 transport services were using servo-control through the use of a Criticool or Tecotherm machine (Figure 6). Neonates needing a transfer for cooling in an additional region were cross covered by a service providing servo-controlled cooling, bringing the total to 15. Four of these transport services modified their existing trolleys to allow incorporation of the Tecotherm device. The remaining were securing the cooling machine (Tecotherm/Criticool) in a fixation trolley separate to the transport trolley. In the 14 services using servo-controlled cooling, all but one of the neonatal transport services currently have a dedicated ambulance. After performing Fisher Exact Testing, teams performing servo-control cooling were more likely to have a dedicated ambulance than teams using a local ambulance service (92% vs 44%, The two-tailed P value equals 0.04).

It is now a nationally endorsed standard to have 24-hour coverage for the transfer of these neonates [4]. In December 2014, all but two of the neonatal transport services in the United Kingdom were also providing a 24-hour service for such neonates. In addition, all services were auditing their temperature control, providing education and feedback regarding therapeutic hypothermia, and using rectal probes to monitor temperature control during transport.

## Discussion

### Therapeutic hypothermia and neonatal transport in the UK

Historically, passive cooling has been endorsed by the TOBY group as the method for providing cooling in neonates with hypoxia-ischaemia who meet the TOBY criteria for cooling [15]. Despite this, Table 1 shows a paradigm shift towards servo-controlled cooling in neonatal transport over the last four years. Currently, 70% of teams use servo-control and 30% passively cool using the TOBY protocol. No team in the UK uses non-servo-controlled active cooling, ice packs, or fans. For teams moving to servo-control over the years, all but one have a dedicated ambulance.

### Servo-controlled cooling versus passive cooling in neonatal transport

A key question is what the literature says about different cooling methods in neonatal transport. Is the move to servo-controlled cooling evidence-based? There is currently only one randomised trial (California Transport Cooling Trial) comparing the efficacy of servo-control versus passive cooling ± ice gel packs in neonatal transport. This was a multicentre trial carried out in California in nine centres involving 100 neonates. Seventy-two percent of servo-control cooled neonates were within a therapeutic range of temperature within 60 minutes compared to 20% passively cooled. Overall, 80% of neonates cooled with servo-control achieved the target temperature compared to 49% being passively cooled ± ice gel packs [14]. The efficacy of passive cooling in neonatal transport described in different studies varies from 20 to 74% [5-13], compared to servo-control, which is between 84-100% [6-8].

There is a higher incidence of overcooling with passive cooling and non-servo-controlled active cooling. Overcooling in neonates being passively cooled varies from 11-34% [5-7, 10-11] compared to servo-control where it is between 0-11% [6-8]. Despite this, 30% of teams are in the UK are using passive cooling. In addition, transport services in Boston and Virginia in the USA use passive cooling with ice gel packs as adjuncts [9-10].

O’Reilly, et al. have proposed that all neonatal transport services should use servo-controlled cooling while transporting neonates with suspected hypoxic-ischemic neonatal encephalopathy [8]. Most neonates with hypoxia-ischaemia are transferred by road in the UK. Long-distance transport makes achieving and maintaining target temperatures a challenging prospect. In the case series of O’Reilly, et al., only 20% of neonates who were passively cooled had temperatures within the therapeutic range, whereas 84% cooled with a servo-controlled machine were within target on arrival at the cooling centre [8]. What is not known is how many neonates being passively cooled started this before or at the time of referral and had rectal monitoring in place to monitor its efficacy. Have units who have moved from passive cooling of neonates to servo-control achieved better results with the latter? A retrospective observational study of infants (East of England) referred to a regional NICU for ongoing therapeutic hypothermia has looked at the results of a cohort of neonates who were passively cooled versus those cooled using servo-control. Of the 134 infants transferred, the first 64 were cooled passively, and 70 were subsequently cooled after the purchase of a servo-controlled mattress. The age cooling was started was significantly shorter in the actively cooled group (46 (0-352) minutes vs. 120 (0-502) minutes; P < .01). The stabilisation time was also shorter in the group who had active cooling. Thirty-nine percent of infants passively cooled were within the target temperature range upon arrival at the regional unit as compared with 100% for infants who were actively cooled.

A study done in Scotland using servo-control demonstrated that in 122 rectal temperatures recorded from commencing servo-controlled cooling, only two recordings (in different individuals) were <33°C. Their experience highlights that minimal staff intervention is needed once cooling is started, allowing freedom to focus on other stabilisation tasks. The reduced risk of overcooling also improves safety [7].

Transport teams continuing to use passive cooling have to reconcile other conflicting considerations in addition to the above evidence. Moving to servo-control is expensive, and there is an imperative to provide a service that is safe for everyone involved. A key part of this is ensuring that all the transport equipment is secure in the event of a road traffic incident and that certain international standards are met. The European Committee for Standardisation has produced certain guidelines (CEN 13976-1:2003 and 13976-2:2003) [16-17]. The recommendation is to use trolleys that have been crash-tested to a European standard in the UK. There is also an emphasis that the weight of equipment excluding the trolley should not exceed 140 kg [16]. In dedicated vehicles, these issues can be overcome by securing the cooling machine separately. However, for teams who may be sending one of many frontline ambulances, it is difficult to make safe arrangements to ensure that equipment is always secure. Even in situations where the cooling apparatus can be secured, the ability of such equipment to tolerate adequate G-forces while accelerating and decelerating is important. This has implications for the safety of both the transporting crew as well as the baby. The risks in terms of a traffic accident cannot be underestimated. There are governance and insurance considerations for staff using equipment that does not meet these standards.  

For some teams, good results may be obtained by teams using passive cooling with minimal side effects. In the study performed by Kendal, et al., 67% of neonates passively cooled during transfer had temperatures within the therapeutic range on arrival to a cooling centre [5]. A study in Boston shows good thermal outcomes with passive cooling achieving a therapeutic temperature in 74% of neonates with only one below the therapeutic range [9]. 

If simple passive methods that avoid the challenges and potential risks of installing extra equipment for transport are effective in achieving therapeutic goals, this may be a sensible way to proceed. Regular audits to ensure benchmarking are important. This study shows a high uptake of audits by all UK transfer teams irrespective of the method of cooling in neonatal transport (Table 1).

### Implementing servo-controlled cooling in neonatal transport

For teams wanting to employ servo-controlled cooling, innovation and adopting new technology is key. Existing transport trolleys can be modified to secure the cooling machine in a secure box within the trolley. This enables use of frontline ambulances. The Tecotherm machine is 7.2 kg compared to the Criticool which is 35 kg. The Criticool or Tecotherm can be secured on a ramp separate to the trolley in the ambulance [6]. For teams who are currently modifying their trolleys, in order to minimise the complication of increased weight and/or to accommodate the servo-control cooling machine, other equipment might need to be sacrificed. Sourcing battery power for the cooling machine is possible in the ambulance but not during transfer from the unit to the ambulance. The availability of dedicated neonatal transport ambulances, which could allow servo-controlled machines to be adequately secured or provide a source of battery power (Figure 4), helps facilitate servo-control. Johnston, et al. in the United Kingdom developed a system [6]. A similar system is in use by the West Midlands Neonatal Transport Service (Figure 1).  Crash testing of systems takes place through a concept called Finite Element Analysis. This technique is used to analyse the stresses, displacement, and ultimately, the factor of safety of the trolley when subjected to ten times the load. This is through computerised testing and simulates the equipment essentially being virtually crash-tested. This survey did not analyse whether all the teams using servo-controlled cooling had been through such a process. For teams wanting to transition to servo-control, this is an important consideration with implications for cost, safety, and insurance.

### Improving the efficacy and reducing complications with passive cooling

A key question is that if servo-control is more efficacious and the risk over overcooling is less, why do some of the teams in the UK continue to passively cool neonates in transport. In this study, six of 21 teams (30%) were continuing with passive cooling in December 2014. When asked why, the reasons provided followed common themes. The use of many front-line ambulances and the lack of a dedicated ambulance was one. Smaller distances with proximity to cooling centres and good results of local audits with passive cooling was another. Other teams felt that the cost involved with the small number of cases being transported for cooling did not justify a move to servo-controlled cooling during transport. For neonates being transferred by air, a helicopter with or without road passive cooling remains the main modality of choice in the UK. It seems logical then that some teams will continue using passive cooling. It must be pointed out that servo-controlled cooling has now been evaluated in cases where multiple modes of transport (fixed wing; helicopter; road ambulance) are used alone or in combination. This is in the United States and the effectiveness is high (100%) [18-19].

For teams continuing to use passive cooling, is it possible to make it more effective in terms of achieving better thermal outcomes? A retrospective study of neonates transported by the University of Virginia showed that clinicians using a fixed protocol for cooling over a four-year period showed annual improvements in the number of babies achieving therapeutic temperatures. Referred neonates were passively cooled, and where necessary, gel packs were used adjunctively [10]. A retrospective study was done to evaluate the efficacy of passive cooling in the Central and Trent region of the United Kingdom showed that early referral and stabilisation were linked with better thermal outcomes. Infants referred within two hours of birth were more likely to reach a cooling centre within six hours from birth (36% vs. 7.5%; p=0.01), with a therapeutic temperature <34°C (50% vs. 0% p=0.02). Infants arriving with a neuroprotective temperature were stabilised longer (median: 143 vs. 97 min p=0.02). Of neonates stabilised for two hours or more, 62% moved from being outside to inside the therapeutic range, or remained between 33-34°C, compared to 21% stabilised for less than two hours (p=0.03). (Sharma A, Leslie A. Factors critical to the success of passive cooling in neonatal transport. PAS archive 2013. E-PAS2013:2840.7)

The development of a regional neuroprotection team in the East of England has helped with early identification and initiation of cooling. Passive cooling was initiated prior to transfer in all outborn infants (n=36) referred between October 2009 and October 2010. During the study period, there was a continuing trend of earlier referral for cooling and earlier achievement of target temperature [12]. The importance of outreach education is nationally recognised [12, 20]. A clear demonstration of its importance is that every neonatal transport team in the UK clearly has tailored education concerning cooling for its local hospitals.

### Cooling in neonatal transport and standards of care

In this author’s opinion, there is an imperative for neonatal transport teams to try and provide the same standard of care for outborn neonates with HIE as for inborn neonates. A recent study demonstrates that the unadjusted rates of adverse outcomes (death or moderate or severe disability; MDI < 70; and psychomotor developmental index < 70) were higher in the outborn population as compared with the inborn population in both hypothermia-treated and control subgroups with HIE. The study failed to show that birth location has an impact on the treatment effect of hypothermia after HIE. It acknowledges that this might have been because of the small sample size [21]. It is clear that amongst available cooling therapies in the NICU setting, non-servo cooling systems are associated with greater variability in rectal temperatures and overcooling compared with servo-controlled systems [22]. There is compelling evidence demonstrating the superior efficacy and advantages of using servo-controlled cooling, which is now being proposed as a standard of care in neonatal transport [23]. That servo-control should be the only method for providing therapeutic hypothermia to neonates during neonatal transport for every transport service around the world must be endorsed with caution. There are clearly considerations concerning equipment, cost, safety, and geography that have to be factored in. The quest for better thermal outcomes has motivated teams in the UK to adopt servo-control cooling. This was a common theme expressed by teams when asked for the reason. Innovation, equipment modification, and dedicated ambulances have helped facilitate this. Teams using passive cooling may argue that there are no direct comparisons of contemporaneous thermal outcomes for infants transferred with either TOBY-passive cooling guidance techniques versus transfers with cooling machines. Such work would inform transport leaders in creating new strategies for cooling infants. It must be acknowledged that the provision of efficacious therapeutic hypothermia to outborn neonates can affect their neurodevelopmental outcomes. The survival and neurodevelopmental outcome of transported neonates who are cooled passively, and those cooled via servo-controlled devices needs to be evaluated as part of carefully designed research trials. The ability to do so prospectively in the UK now could lack equipoise. It is clear that the absence of this data has not, and in this author's opinion, should not delay the provision of servo-control cooling in neonatal transport if it can be delivered safely. Local work in each neonatal network looking at innovative methods of safely incorporating new cooling technology needs to work alongside highlighting strategies for referring centres that ensure the prompt identification, referral, and early initiation of cooling.

## Conclusions

This study highlights that irrespective of the method of cooling used, neonatal transport teams in the UK are benchmarking their outcomes and using technology to enhance the care provided to neonates with hypoxia-ischaemia. There has been a significant shift towards servo-controlled cooling. Servo-controlled cooling in neonatal transport has superior efficacy. Neonates receive servo-controlled cooling in transport with a lower risk of overcooling, and there is potential for it to reduce transfer time. There is an imperative for teams to ensure they can deliver it safely. It is also clear that teams are following a regional approach for identification, referral, and round-the-clock provision of transport. 
